# Enabling uptake and sustainability of supervision roles by women GPs in Australia: a narrative analysis of interviews

**DOI:** 10.1186/s12909-022-03459-8

**Published:** 2022-05-23

**Authors:** B. O’Sullivan, R. Kippen, E. Wearne, G. Wallace, C. Taylor, S. R. Toukhsati

**Affiliations:** 1 General Practice Supervisors Australia, Bendigo, Victoria 3550 Australia; 2 Monash University, Bendigo, Victoria 3550 Australia; 3 Eastern Victoria GP Training, Hawthorn, Victoria 3122 Australia; 4 Melbourne University, Parkville, Victoria 3052 Australia

**Keywords:** Supervisor, Women, Doctor, Medicine, Female, General practice, Family medicine, Primary care, Training

## Abstract

**Background:**

Worldwide, the proportion of women entering careers in medicine is increasing. To ensure diversity and capacity in the general practice (“GP”) supervision workforce, a greater understanding from the perspective of women GPs engaged in or considering the clinical supervision of trainee doctors is important. This narrative inquiry aims to explore the uptake and sustainability of supervision roles for women GPs in the Australian context.

**Methods:**

Qualitative interviews with Australian women GPs were conducted between July and September 2021. Women GPs were selected to represent a range of demographics, practice contexts, and supervision experience to promote broad perspectives. Narrative analysis drew on participant perspectives, allowing emergent stories to be explored using story arcs based on the characters, settings, problems, actions, and resolutions. These stories were evaluated by a broad research team and a high level of agreement of the final narratives and counter-narratives was achieved.

**Results:**

Of the 25 women who enrolled, 17 completed interviews. Six narratives emerged, including: power and control, pay, time, other life commitments, quality of supervision, and supervisor identity. These represented significant intersecting issues with the potential to impact the uptake and sustainability of supervision by women GPs. Some women GPs reported a lack of agency to make decisions about their role in supervision and were not remunerated for teaching. Uptake and sustainability of supervision was constrained by other life commitments, which could be buffered by team-sharing arrangements and a supportive practice. Although adding a burden of time atop their complex and sensitive consultations, women GPs were committed to being available to registrars and supervising at a high standard. To foster high quality supervision, women GPs were interested in up-skilling resources, building experience and harnessing support networks. Women sensed imposter syndrome when negotiating a supervisor identity, which could be managed by explicitly valuing their contribution.

**Conclusion:**

The findings can inform the development of more specific resources, supports and structures to enable women GPs in Australia to uptake and sustain the supervision of trainee doctors at a level they find both acceptable and rewarding.

**Supplementary Information:**

The online version contains supplementary material available at 10.1186/s12909-022-03459-8.

## Background

Worldwide, the proportion of women taking up careers in medicine is increasing, but they are under-represented in leadership roles. Women now outnumber men in medical school graduations in most high- and some low- and middle-income countries [1–3]. In Australia, women constituted 35% of pre-2000 medical graduates, rising to 53% for post-2000 graduates [4]. Women General Practice (GP) supervisors are important role models for teaching and guiding clinical skills development for the next generation of women GPs [5]. Facilitating the uptake and sustainability of supervision for women GPs is further considered important for educational diversity given that women GPs see a different caseload and practice medicine in different ways compared with men [6, 7]. Notwithstanding the clear benefits of women GP’s participation in supervision, there are concerns that women doctors may be less attracted to full time GP roles, along with the time demands of leadership positions and jobs that have unpredictable hours, potentially leaving gaps in the supervision workforce [1, 2, 8–16]. A major issue for maintaining a high-quality supervision workforce is promoting conditions to enable the uptake and sustainability of supervision by women GPs.

In Australia, GP supervisors oversee the safety and training of one or more registrars (trainee general practitioners) whilst concurrently managing individual and practice needs within a private fee-for-service business model. Many practices supervising registrars also host medical students and/or other learners [17]. Within the general practice context, supervision might be viewed as risky because it encompasses person and context dependencies potentially outside the control of the supervisor. Meanwhile, women GP supervisors may have other non-professional responsibilities or interests which intersect with their professional choices [18]. The development of a supervision career may vary between different women GPs relative to the opportunities, structure, and perceived challenges of supervision career pathways.

In Australia, GP training is under major reform to support increased uptake in the general practice speciality, to bolster primary prevention and early intervention services, including for an ageing population [19]. Annually, GP practices and supervisors across the nation host over 5000 registrars at different stages of their training to grow the future general practice workforce [1]. GP supervisors guide the development of real-world general practice skills across the 3–4-year full time GP training cycle [20]. This can be highly rewarding for supervisors who enjoy sharing their expertise and the process of investing in the next generation, however, the specific experience of women in relation to the uptake and sustainability of supervision roles has not been explored [21, 22].

In Australia, as in many other countries, formal (main, lead, or principal) supervisors must be accredited, undertake professional development, and oversee administration of learning and assessment tasks in the practice. Other GPs in the practice without formal accreditation as supervisors may also contribute to registrars’ on-the-job learning, but this may not be formally recognised. Training practices receive a weekly allowance for delivering structured teaching sessions to registrars (1.5–3 hours; ≤ $420 GPT1, ≤$210 GPT2) [23]. This payment is made to the practice, does not reimburse clinical supervision (the oversight of learning on the job such as during consultations), and is generally directed to the supervisor providing the structured teaching session. This may or may not be passed onto each individual GP supervisor contributing to supervising registrars, depending on the decision of the business. Additionally, training practices receive a small weekly training practice subsidy that covers, amongst other things, lost earning for supervisors whilst they are teaching or observing registrars and are not seeing patients during in practice teaching (≤ $560 GPT1, ≤$280 GPT2) [23]. Once again this may not be passed on to supervisors.

In Australia, GP registrars are employees of the practice in which they train and conduct fee-for-service patient consultations. This contributes to practice income and helps to keep the teaching costs (lost billing time for supervising) tolerable for the business, including where high quality supervision takes time [24].

Limited available research has explored the degree to which the current systems and processes around the supervision of GP registrars in private general practices align with the needs and interests of supervisors. One study identified that rural women GP supervisors were less likely to participate in supervision than were men, but this effect diminished once adjustments were made for total doctors employed in the practice, business relationship with the practice, and total hours worked per week. This suggests that women’s participation in supervision is likely to intersect with multi-level demographic and practice contextual influences across the lifespan [17].

The broader literature denotes that there may be gender bias in medicine that leads to stereotypical responses to tasks and roles that can impact power, and economic and social prosperity [25]. Women GPs may frame their work identity to conform to gendered expectations despite this playing out negatively for their financial and professional status [26]. These issues may present challenges for supervision roles within general practice training. Research is needed to explore and understand the narratives of women GP supervisors, currently supervising or not, to better inform fair and inclusive environments for women GPs to become supervisors. This narrative inquiry aims to explore GP supervision in Australia from the perspective of women GPs to inform how to engage and sustain women GPs in supervision roles.

## Methods

### Study design

Qualitative interviews were used to explore the perspectives and lived experiences of women GPs in Australia around supervision of GP registrars [27].

### Participants

The research team received interest from 25 women GPs in Australia, from which 17 women were purposively selected to ensure representation across a range of practice roles, personal circumstances, and supervision experiences—currently supervising, previously supervising, or had never supervised, which are characteristics known to occur within a constant dynamic [28].

### Procedure

Ethics approval to conduct the study was granted by the Monash University Human Research Ethics Committee (# 28848) on 28th May 2021. General Practice Supervisors Australia (GPSA)—Australia’s peak body advocating for GP supervisors—emailed an invitation to their membership list of around 5500 individuals, for women GPs to participate in the study. Members were requested to share the invitation with other women GPs who might be interested in participating. Potential respondents registered an expression of interest online which collected basic demographic and practice data.

The interview schedule (see Additional file 1) was piloted with the research team and five women GPs known to the research team, and refined to explore participant stories, including issues related to the uptake and sustainability of GP supervision roles. One-to-one semi-structured interviews were conducted online between July and September 2021. Each interviewee provided written consent to enrol in the study and was given an AUD $150 gift voucher in recognition of participation. Interviews were recorded and transcribed verbatim.

The interviews were conducted by two non-GP health services researchers employed by GPSA, with the aim of exploring women’s experiences to inform the development of appropriate resources and policies. The interviewers had no preconceived notion of what the women’s stories might be, as there is no other research about this topic. Neither researcher had been a GP supervisor, and they did not know any of the participants. There was no specific gender lens applied to each interview due to the intersectionality of gender with other forms of inequality and exclusion [29].

During the interviews, the interview schedule was used as a guide, with extemporaneous evolution allowing the women GP interviewees to discuss and expand their own narratives without interruption. The interviews ceased at the discretion of the interviewees, when they had nothing more that they wanted to add.

### Data analysis

Narrative analysis was used to elucidate participant perspectives, allowing different stories to emerge and be arranged for their meaning to inform the topic. After each interview, reflective notes were recorded to develop initial impressions of the stories of each woman, and these were discussed by the research team to inform deeper reflection. The researchers fully immersed themselves in the data by reading and re-reading the transcripts over the course of 3 months and discussing the main stories that were emerging.

The researchers sought to represent the legitimate meaning of the women’s stories as part of the narrative analytical process and reduce any subjective biases [30]. Data analysis focused on re-storying women GPs’ experiences of registrar supervision through creation of story arcs, which reflected the everyday practical experiences of each participant [30]. Key elements from each narrative were identified comprising characters; setting; problems; action; and resolution (see Additional file 2). These were then arranged in chronological order [30]. The interpretation of each re-storied narrative was repeatedly discussed amongst the research team. The temporal unity and complexity of the data were protected to relate the lived experience of women GP supervisors and reflect on the capacity for women GPs to take up and sustain registrar supervision. Where counter-narratives emerged, these were also documented to provide for richer interpretation.

## Results

Of the 25 women who completed expressions of interest for the study, 17 participated in interviews representing almost 17 hours of recorded data. The sociodemographic characteristics of the sample are presented in Table 1. In summary, a range of characteristics were represented, although most respondents were aged under 45 years, were partnered, had children or were expecting, and were currently supervising. There was a similar level of representation across those working part and full time and working in a range of practice sizes.

**Table 1:** Sociodemographic characteristics (*N* = 17)

Characteristics	Number
***Age group***	
Under 45 years	11
45+ years	6
***Partner***	
Yes	14
No	3
***Children***	
None	6
Pregnant with first child	2
Youngest child < 5 years	2
Youngest child 5–17 years	4
Youngest child 18+ years	3
***Work status***	
Full time	8
Part time	9
***Supervision status***	
Never been a GP supervisor	2
Currently a GP supervisor	10
Previously been a GP supervisor	5
***Experience as supervisor***	
None	2
In a team (informal role)	6
Formal (main listed supervisor)	9
***Number of GPs in practice (full or part time)***	
1–5 GPs	6
6–10 GPs	7
11+ GPs	4

Six intersecting story arcs emerged from the qualitative interview data which were about power and control, pay, time, other life commitments, quality of supervision, and supervisor identity. These are summarised in Table 2.

**Table 2:** Summary of story arcs and descriptions (*N* = 17)

Story arcs	Description
Power and control	• Women GPs describe having limited control over the decision to supervise registrars and were not fully informed about the role. • Some women GPs play a key role in the formal and informal supervision workload in practices that is not appreciated as a valued contribution. • Some male registrars may disrespect women GP supervisors as mentor-teachers. • Some women GP supervisors felt unsupported by male superiors to manage male registrars who were not receptive to their feedback. • Some women GPs lead the teaching and learning in their practice, but, if engaged as a non-practice owner, they may not get adequate practice support to sustain the task.
Pay	• Some women GPs describe being unaware of and unremunerated for aspects of the supervision role. • Women GPs can experience unsuccessful negotiations with male practice owners around pay for the structured teaching they did. • Women GP practice owners and non-practice owners with lead educational roles in the practice are inclined to remunerate women GPs for structured teaching. • Inadequate remuneration relative to the workload can affect early-career GPs interest in doing supervision work and mature GPs from continuing it. • Pay is valued as part of recognition by women GP supervisors of different demographics and career stages.
Time	• Women GPs believe they are sought out by registrars because they are approachable and value registrar learning and well-being, regardless of whether supervising formally or informally; but the time needed for frequent encounters is frustrating for women GPs when they are not the main supervisor. • Women GPs are asked to support registrar learning across women’s health, mental health, sexual health and complex care where they are perceived as experts, and this interrupts the time they need for their own patients (sensitive consultations). • The time commitment for supervising is worse if registrars are junior, unsafe or under-performing. • When women GPs take a break from or relinquish supervision roles, they express a sense of relief at not having to worry about learners and having time to do other things, such as invest in their own learning.
Other life commit-ments	• Women GPs of various ages describe the challenge of managing personal commitments, particularly to parents and children, with committing to supervise registrars over a 6-month term. • Informal supervision roles allow women to accommodate other life responsibilities whilst enabling them to be involved in supervision. • Women planning to have children describe potential career disruptions as a barrier to supervising. • The capacity to juggle children can vary depending on how family-friendly the practice is, and the proximity between practice and childcare/school. • Overall, women GPs may view supervision as an additional effort atop of their professional and personal lives.
Quality of supervision	• Women GPs are intrinsically motivated to provide quality teaching and learning to create a positive experience for registrars. This could deter women from supervising unless they felt able to do it in a way that met their personal standards. • Women GPs actively pursued ways to build their supervision expertise to enable them to supervise to a high standard. To this end, women GPs noted a lack of educational support and guidance to foster understanding of the supervision role. • Women GPs preferred team supervision to provide backup for registrars and opportunities to share ideas. • Women GPs gained confidence from teaching medical students and overseeing registrar learning in other general practices (as an independent clinical educator). • Women GPs seek formal and informal opportunities to exchange ideas and share resources with other GP supervisors, such as supervision mentoring. • Women reflect on and reconcile the level of uncertainty involved in supervision which enables them to keep supervising even when registrars don’t progress.
Supervisor identity	• Imposter syndrome is common in women GPs commencing supervision roles. • Early-career women GPs think they lack sufficient technical GP knowledge to be teaching, but perceive strength in their fresh knowledge of the GP training program and are encouraged if they are in a supportive team where they can learn to supervise and their value to the team is acknowledged. • The historical requirement for early-career GPs to get some experience before taking up supervision roles was viewed as a barrier to new women GPs to take up supervision. • Some mid- and later-career women GPs experience imposter syndrome if they lack knowledge of current GP exams and clinical guidelines, but they draw confidence from their expertise in real-world practice, which helps them be assertive about their value. • Women GPs overcome imposter syndrome and build confidence in a supervisor identity when they can bounce ideas around a team, reflect on their practice, and realise their unique contribution is based on the types of patients that they see, the way that they teach, their specialisations, and the nature of medicine that they practise.

### Narrative analysis

A description of the narrative for each story arc is presented with exemplars below.

### Power and control

Several women GPs working as non-practice owners described having been asked to take on supervision without being fully informed, and at times being misinformed, about the role. This pattern had the potential to repeat as women GPs moved between practices.

*I fell into it, so it actually happened when I was working in [regional centre], and the practice manager just handed a form and said, "Can you sign this, because we need an extra person to supervise?" And I think I did say something like, "So long as you're not expecting me to actually do anything," she said, "No, we're not," but of course they were. So that's how I sort of fell into it, and then…, when I changed jobs, one of the things they said [in the new practice] is they need a supervisor, which was fine. I didn't realize that they actually needed a primary supervisor, I didn't realize that that's where that was heading. So, that's how I got into GP supervision. [ID1]*

One woman GP, working in the same practice for over 20 years as a non-practice owner, related a similar story of being nominated as an official supervisor on formal paperwork without her consent:

*I actually didn’t put down my name to actually be an official … supervisor. Although I was teaching, I was doing it in an unofficial manner. But my boss [practice owner] took it on himself with his wife to forge my signature to say that I was going to be prepared to be doing this teaching. One day, three registrars arrived. [ID8]*

She found this frustrating because her efforts to accommodate the situation were not acknowledged:

*…I had my nose a little bit out of joint, because [practice owner/boss] didn't pay me anything, I didn't get any thanks. It was just sort of assumed, okay, well now you've agreed to it, goodbye. [ID8]*

When asked to supervise again later at the same practice, she sought more control over the process including asking for payment *- “I’m going to actually speak to [practice owner/boss]. And so I asked him to pay me…” [ID8]* - but this resulted in her being excluded from ongoing supervision opportunities:

*…when the next one came without telling me I suddenly did not become the supervisor and I haven't been the supervisor since. [ID8]*

She subsequently only supervised informally, disjointed from the formal supervision team but contributing in a way that she had control over.

Women GP supervisors related doing a substantial proportion of informal supervision without recognition or authority:

*I would be sitting in a room with a junior registrar, the GPT1 in the next room on their first two weeks, and on the weekends, because I worked a lot of Saturdays. And there'd be no recognition. There wasn't even a thank-you for doing it. And that didn't make me want to give it up, but it made me really [upset]. [ID2]*

Several women GPs also told of having a lack of power in relation to overseeing male registrars:

*I wondered sometimes if he just culturally struggled with having female supervisors. I often felt like he didn't listen or take things in as much from me as he might've from my male colleague. [ID5]*

Another woman GP supervisor sought help when she felt that a male registrar was not receptive to her feedback and advice, but the situation remained unresolved due to a perceived lack of empathy from male superiors:

*I remember telling … [one of my] superiors about it... I was trying to explain to him the trouble I was having with this registrar, and how he wasn't receiving feedback. But this other, this contact person, was also a male GP, and I don't think he really fully understood that. He kind of just brushed it off. [ID11]*

The counter-narrative about lack of control and power for women GP supervisors was seen to develop where women GP supervisors who were non-practice owners led all aspects of supervision for their practices and were acknowledged for doing so. These women had strong agency and enjoyed building a supervision model:

*I just did it all... everything, including all the admin of it and getting all the registrars and all of the supervising of the registrar. So it was the only way I could get it running…if I just did it… my purpose with that was about making [supervision of registrars] sustainable. [ID9]*

However, there was not practice support to sustain the teaching and learning model this GP had planned:

*they just didn't want to be involved. [ID9]*

### Pay

Some women GPs who were working as non-practice owners had not questioned why they had never been remunerated for supervising. When other women GP colleagues alerted them to the availability of funding for teaching, they approached male practice owners to talk about getting paid for the teaching aspect of supervision work:

*I didn't even know that practices or supervisors were being remunerated… one other colleague [senior female GP] said, "Oh, don't you know that they do get paid?" I was like, "Oh, no idea," they're like, "You should ask."[ID6]*

Among others, one mid-career woman GP working as a main supervisor and a non-practice owner, approached the practice manager about not getting paid:

*I just wasn't getting any answer. And I was going on more and more teaching… I phoned up the owner, just to try to talk to him directly, because I wasn't getting any sense from the manager, and the owner said, "I've got to make an income, those payments are mine. You should do the training in your own time." [ID4]*

She was unsuccessful in the negotiations and ended up leaving the practice feeling unappreciated:

*… one of my colleagues said, "… I think this is time for us to leave." And so, both of us, we decided to move to a practice that appreciated teaching and that appreciated we weren't just magically becoming GPs and there was a point of teaching. [ID4]*

Other mid-career women GP supervisors, working as non-practice owners, added to the story that, over their careers, they had become more willing to advocate for remuneration of their teaching in supervision work or relinquish it:

*I think when all you do is work in general practice and you're not remunerated for your contributions for teaching, but your income drops because you're providing supervision and teaching… I wouldn't accept that now, whereas in the past I just accepted that as the way it was…now I'm old enough and cranky enough, I just say, "No, sorry, can't help you." [ID10]*

Countering this narrative, some women GPs who led teaching and learning in their practice as practice owners or educational leaders (working on a contract) recounted that they ensured supervision payments were distributed to the GPs who were teaching:

*...it has been the practice principals who have been doing the teaching most of the time, but if we have had someone, one of the other contractors [non-practice owner] doctors doing a teaching session for us, we have always given them the equivalent share of the teaching payment. We feel like that's only fair. [ID5]*

However, even for those receiving payment for supervising, the payments were considered small relative to earnings that women GPs could make from billings:

*...when I spent the morning sitting in with my registrar yesterday, I sort of got my normal supervision payments but essentially that is nothing compared to if I was seeing my own patients, because the registrar's done all the billing for that… if it wasn't a financial disincentive, that would be nice. [ID17]*

One early-career GP noted pay was a necessity given the time she spent on supervision:

*I think the income support actually was a big factor…I perhaps was a bit naive about how much time it would take, especially because I want to do it properly. [ID13]*

Pay was also an issue for a mid-career woman GP, particularly in relation to choosing work which did not disadvantage her for working part-time and having had more breaks in practice:

*I did want to get paid for what I was doing. I think that the finances do come into it; I think it's got to be adequately remunerated… women tend to work less hours and have time off, so that might be more of an issue than for a man possibly. [ID8]*

Women GPs who were single and/or co-parenting also reflected that the pay is important as part of a portfolio of income that they relied upon to live; and if the pay for supervision was inadequate it added to the reasons to cease supervision:

*[leaving supervision] …it was just a combination of … income pressures, and also a registrar that's just not appreciative …. I just decided, "No, I can't do it. I just have to look after myself." [ID4]*

### Time

Many women GPs identified with being approachable and available as supervisors, but noted that this increased the time they dedicated to supporting registrar learning and well-being, whether or not they were the main supervisor:

*They know that they can call me at any time. I have no hesitation, I'd rather them call me than not. [ID3]*

One woman GP noticed that a group of her early-career women colleagues were frequently used for informal support because the main male supervisor was not considered approachable:

*…the [official] supervisor was male, and then there were at least three of us that were female …the interesting comment was that "Oh yeah, you guys are more approachable, you guys are more accessible. It's easier for me to come and ask you questions and ask for some advice as opposed to going to my supervisor." [ID6]*

They tried to resolve some of the boundary issues with the registrar and ensure the main supervisor was accountable to his role. However, the situation was not resolved, and this eroded the women GPs’ time with their own patients whilst they supported the needs of the registrar. This caused frustration:

*...it's typically not part of our job description … We don't mind, helping out here on the odd occasion, but if suddenly someone is calling you 5, 6, 7 times in a session … It does take out time from your own practice. [ID6]*

Women GP supervisors also discussed being frequently called upon for advice on issues for which they were perceived as experts and these could be the more time consuming cases:

*…They don’t have 6 problems, they have like 20, and there are like 35 tablets. Apparently, I’m the juggler of that. [ID16]*

Women GPs described fitting supervision around a broader scope of medicine they practised:

*… our consults can be more complex and time-consuming compared to some of my male colleagues... It can be hard when you're talking to someone about a mental health issue…then you're kind of interrupted to talk to a registrar to do teaching. It can get quite awkward. [ID15]*

The time that women GPs dedicated to supervision increased if registrars were junior, unsafe, and/or under-performing:

*...we haven't had a [first or second 6-month term registrar]… for a while and they're always the ones that are very time-consuming … they ring you a lot, then that's exhausting. [ID5]*

This scenario led some women GP supervisors to consider giving up supervision work:

*We've certainly had in the last couple of years, a few... a couple of tricky registrars, and this practice has been taking registrars for probably 25 years. And the staff are exhausted [ID5]*

When women took a break from supervising, they felt relief:

*It gives the time to do other stuff... I'll sit down and do some other things as well, rather than having to worry about where the learning needs are.... more time to just do my own learning. [ID5]*

### Other life commitments

Women GPs reflected on both unpredictable or fixed commitments in their personal lives and how supervising over a fixed learning term would align with this:

*Outside my personal things and my work… knowing that if you're going to commit for an official thing [supervising], you're committing for six months. And so, again, I would hate to take on a role and then say, after three months, “oh, sorry, I can't do that.” [ID8]*

Other women GPs yet to start families anticipated major breaks in practice when doing this, which could disrupt supervision momentum:

*For me obviously, now I'm going to have a kid. Then go out, search what I need to... Work out what I need to do to fill out the paperwork, go to the training days. Those kinds of things I think will make it harder for me, definitely… [ID7]*

There was a sense of unpredictability related to raising young children which could make it harder to supervise:

*...that is a significant thing to think about, having children. And it is something that I don't think male …GPs and male supervisors have to really think about as much…the hard part is the unpredictability of it. [ID11]*

Primary school-aged children were noted to impose scheduled demands which were difficult for women GPs to manage around supervision tasks, including completing paperwork:

*I've been more conscious since having a family... You've got more deadlines. You've got to finish on time, to get home for children. Previously, it didn't matter if I worked late or stayed late to do paperwork. But it matters now. [ID12]*

However, the capacity to juggle children around supervision roles improved if women were in a supportive and flexible practice and the childcare supports were nearby:

*...the practice has always been really family-friendly, …really good at juggling. My babies always came to tutes with me and stuff like that, it was never an issue for clinical meetings. [ID17]*

However, this supervisor still reported that supervision took an additional effort: *even if we have someone at home, juggling kind of the family life and that sort of thing, and needing to be home for kids or go to other things, sometimes it’s just an extra thing to do. [ID17].*

### Quality of supervision

Women GPs related stories which showed they were intrinsically motivated to provide quality teaching and learning to create a positive experience for registrars. Correspondingly, they were reticent to get involved in supervision unless they could meet their personal standards, overlapping with the theme about their other life commitments:

*I don't know that I would want to go into being a supervisor if I can't do it properly. If can't do it properly, can't do it well, then what's the point? [ID7]*

Women GPs related a belief that best practice meant going above and beyond the minimum requirements by developing and nurturing the supervisor-registrar alliance:

*I probably do a bit more than what is prescribed, I guess, [or] expected. And a part of it is because I think it's really like I value registrars and I think that they should get a really good experience, and part of it is for me as well: because I want to make sure that I'm comfortable. [ID3]*

Women GPs aimed to build skills and find the right practice culture to foster their capacity to supervise to a high standard:

*...if I was in the right environment, yeah, I would consider being a supervisor…[and] do the training. [ID7]*

However, women GPs’ stories indicated a lack of specific educational support and guidance to enhance their understanding and benchmarking of the supervision role, which led these women to search for information themselves:

*I don't remember doing any specific education on how to be a supervisor. I had to figure out a lot of stuff myself or by asking other people. [ID7]*

This imposed a level of responsibility for women GPs commencing supervision such that *others may not know about the process, or they don’t know what’s involved, and they might feel this sense of burden or responsibility because they don’t know to what level they’ll have to have to start supervision. [ID15].*

Women GPs actively pursued ways to develop their supervision skills by reflecting on their own experience as a supervisor and through teaching medical students, or benchmarking from being involved in external clinical teaching visits (ECTVs):

*I feel like I've built up more skills over time, with teaching and training and learning and feedback. [ID12]*

*I want to be involved…to do ECTVs, to make sure that I... build my skills in supporting registrars. [ID13]*

Women GPs identified the need for backup support for registrars, showing preference for working as part of a team of supervisors:

*… it's nice to have other colleagues there... if I'm not there, there's two other people…[so] I know that the registrar's not alone…. It's also nice to have another supervisor or two to discuss feedback and your thoughts… just to bounce things off others. [ID3]*

She also sought wider opportunities to exchange ideas with GP supervisors from other practices.

### Supervisor identity

Women GPs related stories of having imposter syndrome when they started supervising. This was underpinned by a lack of confidence about their technical knowledge of being a GP:

*I think a lot of female GPs worry that they don't know enough to supervise or to teach… they think they do have to teach that really technical knowledge… the old imposter syndrome. [ID17]*

This concern was generally reflected by woman GPs who were early in their career:

*…you have that imposter syndrome. I wasn't sure, I was only three years post-fellowship and I thought, "What am I going to teach these guys?” [ID3]*

One woman GP in early-career noted that this was heightened at the thought of being a main supervisor:

*...we've only had one registrar since I've been officially a supervisor… I was a little bit terrified anyway, to be their main supervisor… [ID13]*

This woman GP supervisor worked in an education-focused practice with very experienced senior supervisors, which made her question her supervision identity:

*I don’t know if it was just the caseload, or just me learning how to be a supervisor, as the challenge. [ID13]*

She felt the feedback about her genuine value to the supervision team played a part in legitimising her contribution, *…they think I’ve got things to offer with the topics or experiences and style that I have… they think I’ve got something to give*. And it also helped that she was supported to learn the supervision role by other senior supervisors rather than coming in as a supervisor expert:

*I was quite intimidated initially and I'm like, "I have no idea what I'm doing." I still feel that, but …knowing that I'm going to be taught how to teach has helped. [ID13]*

Some women GPs wanted to begin supervising early post fellowship because, at this time, they had fresh knowledge of the training and assessment processes:

*…as a new fellow, you kind of know the system, you know the exam process, you know you're pretty up to date with all that kind of thing. Probably if it was easy enough to have done it, I would've done it probably straight away… I think it's a bit harder now... Exams have changed… I am no longer up to date... I don't have those resources anymore... [ID7]*

Mid- and later-career GPs also described personal experiences of imposter syndrome. This was based around lacking confidence about GP exams and current clinical standards. Women GPs overcame imposter syndrome about this perceived knowledge gap by reflecting on their real-world knowledge and their capacity to role-model work-life balance:

*...there's no point me teaching them about heart disease or how to read an ECG because his being a cardiology registrar, he's better at that than I am anyway. But I can teach more about the art [of being a GP]… I try to do role modelling… balancing kids and work. [ID17]*

Mid- and later-career women overcame imposter syndrome by *having a couple of people who are dual supervisors and can bounce stuff off each other*… *just helping with that confidence [ID17]*; and using self-reflection to build comfort with not having all the answers:

*I've got enough self-checks on myself to not doubt myself too much…. I’m not pretending to be anything more than what I am… I'm quite happy as a supervisor to say, "I don't know either." [ID16].*

Women GPs also reflected on their unique style of medicine, types of patients, and the style of teaching they experienced in their own training for building identity as a supervisor:

*… there's definitely male general practice medicine which is quite different to the things females see for the most part. Style of teaching I think varied a bit too, for me anyway, in terms of female and male. [ID7]*

Women GPs’ stories reflected that they became increasingly assertive about their value as they matured in their career:

*It's only now that I'm quite old that I can actually behave more like a wicked witch and stand up for myself..” [ID14].*

There was a perception that the next generation of women GPs would likely be more assertive:

*... I think [the next generation of women] … have less barriers because they're all more assertive…than maybe my generation was, or maybe I am. [ID2]*

## Discussion

The aim of this narrative inquiry was to explore the perspectives of women GP supervisors in Australia to facilitate diversity and capacity in the GP supervision workforce. Our findings suggest that there are intersecting experiences which could underpin women GPs’ willingness to take up and sustain supervision roles. Women GPs in general practices who are non-practice owners may lack agency in business decisions related to supervision, such as formal recognition for supervision and payment of teaching allowances. Similarly, stories from early-career women GPs speak to a lack of recognition and remuneration for teaching, which can deter them from taking on supervision in the future; mid- and later-career women GPs shared these experiences and opted to cease supervision unless they were given recognition and remuneration for paid components of the role.

These findings are consistent with the extant literature about inequality regimes arising from complex, systemic, and interlinked inequalities in workplace practices and processes [31]. We found that women GPs commonly contributed to teaching and supervision work in informal ways that benefited practices. However, this labour was often hidden and without pay or recognition, despite the availability of remuneration for teaching and women GP supervisors placing a premium on the quality of their supervision, as well as their availability and approachability as supervisors. The investment that women GPs make in supervision, albeit sometimes without recognition or reward, is likely motivated by interests to protect the safety of learners in the practice and to foster trainee doctors interested in working as GPs and in the practice in the future. However, it also suggests a gendered substructure to practice supervision, where inequalities are built into women’s roles and women supervise around a set of explicit and implicit rules related to gender and their position within the practice [29]. An important issue of gender equity within contemporary medicine is ensuring that female GPs, particularly those in early-career, have access to information about supervision roles within different practices, payments for supervision relative to the different roles, the process to get involved, and access to a support network such as a peak body. It may also benefit women GPs if supervision policies require that practices consult with women GPs for their inclusion in supervision teams, documents the roles of supervision teams, and provides a clear statement about supervision activities that are eligible for remuneration.

Women GPs in this study also reported being asked to support the teaching of sensitive topics like women’s health, mental health, and sexual health. While this provides diversity in practice teaching, it can also create tensions for women GPs trying to navigate caring for their own patients and managing registrar interruptions. Despite their value as supervisors, women GPs expressed a lack of confidence in supervising and seek validation through their contributions to technical or real-world medicine that they bring to the role. It is possible that this belief relates to women fitting into the socio-relational context of medicine as an historically male profession and assuming a gendered role which reinforces inequality [32]. The respondents generally believed that the next generation of women GPs would break through this stereotype and be more assertive; however, this will largely depend on better acknowledgement of the valued contribution women GP supervisors might make at all stages of their career. Confidence could also be supported through more woman-specific mentorship networks for supervision. Such professional networks could be useful at practice, regional, state, and national levels, and might assist women GPs to develop their supervision skills and confidence and improve awareness of and access to useful resources. This aligns with other research suggesting the need for effective and sensitive professional development and accommodation of different work patterns as the GP workforce is feminised [33].

A major consideration for policies seeking to enhance women GPs’ uptake and sustainability of supervision is enabling women to enter and re-enter supervision roles across their career span. Australia remains relatively wedded to the normative, unencumbered, male worker archetype which relegates women to juggling paid and unpaid work [34]. However, many GPs also choose not to exclusively focus on work, which is aligned with research that suggests generalist doctors are motivated by social and family values as part of their career choices [35]. As a result, supervision roles in general practice need to accommodate both professional and (unpredictable and fixed) non-professional roles that women, and men, adopt across their life course. A key option to manage this is to promote team-based supervision where women GPs have a clear role and can make a quality contribution through a shared commitment. This may allow women to develop and retain their identity as a supervisor for longer, and to leave and re-enter supervision if they take breaks from work or work part time.

This research was exploratory and was limited to Australia, but it is the first to explore the lived experience of women GP supervisors. The study was well-subscribed by women at various stages of their GP supervision careers, providing a rich opportunity to reflect on different narratives. We acknowledge, however, the possibility that more interviews could have exposed a wider range of stories. Australia has a unique GP training system and women GPs may have different experiences in other countries. This research was designed to explore the stories of Australian women GPs and, although it did not take a gendered lens, the findings do relate to feminist theory. It will be important to expand on this research to explore male GP supervisor experiences to confirm whether a gendered interpretation is valid.

## Conclusion

This research expands understanding of the lived experience of Australian women GP supervisors as they navigate taking up and managing supervision roles. The research points to story arcs which were about power and control, pay, time, other life commitments, quality of supervision, and supervisor identity. These represent significant issues that intersect to potentially impact the interest and capacity for women to join and be retained in the GP supervision workforce. The findings can be applied to developing more specific resources, supports, and structures to enable women to participate in and sustain GP supervision at the level that they find acceptable and rewarding.

## Supplementary Information

**Additional file 1.** Semi-structured Interview guide. Tabulated semi-structured interview questions and prompts.

**Additional file 2.** Story arc framework. Tabulated story arc framework and description.

## Data Availability

The datasets generated and/or analysed during the current study are not publicly available due to ethical restrictions but are available from the corresponding author on reasonable request.

## Abbreviations

GP: General practitioner
GPSA: General Practice Supervisors Australia
GPT1: General Practice Term 1— 1st 6-month training term for GP registrars
GPT2: General Practice Term 2—2nd 6-month training term for GP registrars
KFP: Key Feature Problems
