# Supplementary material for: Enabling uptake and sustainability of supervision roles by women GPs in Australia: a narrative analysis of interviews
Source: BMC Med Educ. 2022 May 23;22:398. doi: 10.1186/s12909-022-03459-8 (PMC9128131; doi:10.1186/s12909-022-03459-8)
Supplement: Supplementary file 1 — Additional file 1. Semi-structured Interview guide. Tabulated semi-structured interview questions and prompts. [file 12909_2022_3459_MOESM1_ESM.docx]

**Additional file 1:** Semi-structured Interview guide

| **Question** | **Prompting frame** |
| --- | --- |
| Tell me a bit about yourself as a GP and your practice? | Practicing, your employment there (salaried etc.) any areas of interest, current FTE (has this changed over time)  Your practice – location, type size  Your background as a GP  Your experience of being supervised |
| Tell me a bit more about yourself as a person | Any familial or social roles |
| What is your current experience regarding supervising registrars? | Or your experience in the past? |
| When did you start supervising and how did you start? | Or when do you plan to start supervising? Why? |
| Why did you start supervising? | What motivated/s you/the triggers to start? |
| Can you describe your supervision role when you started? | Did you have any concerns when you first started supervising?  If so, how did you manage these? |
| What did/do you enjoy about supervising? |  |
| Since you started supervising has your role in supervision changed? | If so, how and what triggered that? |
| Have you taken any breaks in supervision over time and why, why not? | If so, what was your experience of not supervising over that period? |
| If you took a break, were you concerned about coming back to supervising, and why, why not? | How easy was it to come back to it? What would help you to come back? |
| At a broad level, what is your preferred approach to supervision? |  |
| To what extent have you been able to supervise in this way? |  |
| Overall, has there anything as a female GP supervisor that has been challenging and why? |  |
| Is there anything that has made you want to give it up/ made you give up supervising? |  |
| What has helped you keep supervising over the period you have been doing it, and why? |  |
| Thinking more broadly now, what barriers do you see for women GPs to supervise registrars in general practice? |  |
| Again, more generally, what do you think might help women GPs to supervise registrars? |  |
| Is there anything else that I haven’t asked you about, that you would like to add? |  |
