# Supplementary material for: Enabling uptake and sustainability of supervision roles by women GPs in Australia: a narrative analysis of interviews
Source: BMC Med Educ. 2022 May 23;22:398. doi: 10.1186/s12909-022-03459-8 (PMC9128131; doi:10.1186/s12909-022-03459-8)
Supplement: Supplementary file 2 — Additional file 2. Story arc framework. Tabulated story arc framework and description. [file 12909_2022_3459_MOESM2_ESM.docx]

**Additional file 2:** Story arc framework

| **What** | **Description** |
| --- | --- |
| Characters | Information about the individuals involved, their archetype, personality, behaviours, style, patterns. |
| Setting | The place or the practice, conditions, time, locale, year, and era. |
| Problems | The question being answered or phenomena to be described or explained. |
| Action | The cognitive actions and movement or attempt through the story, intentions and emotional drivers or goals of the characters, include failed and successful attempts. |
| Resolution | Final answer to the question and what caused the turning point. |
